# Practical Departments

**Published:** 1901-12-14

**Authors:** 


### PRACTICAL DEPARTMENTS.

### AMBULANCES AND STRETCHERS.

(Messrs. Wilson and Stockall, Bury, Lancashire.)

Messrs. Wilson and Stockall, whose ambulances gained such distinction at the Paris Exhibition of 1900, have just issued their new catalogue, showing the latest developments in arrangements for the transit of sick and injured persons. Some very excellent and useful suggestions from medical officers of health, surveyors, officers of ambulance associations, and others have been adopted. Messrs. Wilson have just built two horse ambulances for the Corporation of Manchester. These are designed for the removal of two patients at a time, and the most noticeable feature is the easy provision for ingress and egress, the carriage being hung so low on a cranked mail patent axle that an injured person can be placed in position with the greatest possible ease; and owing to a particular mode of adaptation of the springs on patent rubber ball bearings, any jolting or uncomfortable motion is reduced to a minimum, the wheels being rubber-tyred. The ambulance contains two stretchers, one of which is slung and runs in on a folding frame with a special patented arrangement for raising and lowering. The second stretcher is furnished with 12-inch rubber tyred wheels, elliptic springs and axle (easily detachable), and can be used to run a patient along the floor of the works or mill, or carried up and down stairs; it runs on grooves into the carriage from the portable tramway, which is fitted to the back of the ambulance: this neatly folds up, is carried with the stretcher, and is always ready. The stretcher is also fitted with an ingenious arrangement for raising the patient when descending a steep incline. Both stretchers are covered with stout sail canvas and movable rubber sheeting, and two pillows are supplied. There are two lamps, one outside and one in, a brake, shafts, and plate-glass embossed side windows; the body is varnished self-coloured wood; the under works and wheels are painted and very highly finished. The second stretcher runs on springs like those of a highly finished perambulator, and these, with the wheels, are easily removed, so that the stretcher may be used equally well for carrying the patient; and, on the polished floors of a hospital ward, it could be used for the transit of patients to or from bed.

A special brougham ambulance is made for removing infectious and sick cases. This may, if required, be used as an ordinary brougham to seat four persons; there is a small cupboard for medical accessories, and the outside is painted like an ordinary brougham; the inside, being highly varnished, can be washed after use. It is fitted with a portable stretcher which is made with Dominion patent wire mesh and spiral springs: this runs in on a grooved frame which will extend, and then neatly folds up out of the way when not required; the whole of the frame and stretcher can be removed in a minute.

Other and cheaper ambulance vans are also shown, and Messrs. Wilson claim that their "new registered disinfecting van" is the most convenient vehicle as yet produced for the removal of infected bedding, clothing, etc. The doors at the back open outwards; the interior is lined with zinc, and all the joints are soldered; a metallic sliding shelf is provided in order to avoid as far as possible personal handling of the bedding; there is a portable seat at the back for the inspector, and there are two seats in front.

A useful four-wheeled litter is designed for removing patients from their beds to the operating theatre: this is made to suit the height of the operating table. Another ward litter has the wheels placed inside the springs, so that there are no projections at the sides.

A most useful little appliance is the "bandage shoot," for hanging on the wall: it is used by the St. John Ambulance Association, and by several of the largest railway companies, and contains materials for first aid in cases of accident.

